# Have dipeptidyl peptidase-4 inhibitors ameliorated the vascular complications of type 2 diabetes in large-scale trials? The potential confounding effect of stem-cell chemokines

**DOI:** 10.1186/s12933-017-0648-x

**Published:** 2018-01-08

**Authors:** Milton Packer

**Affiliations:** Baylor Heart and Vascular Institute, Baylor University Medical Center, 621 N. Hall Street, Dallas, TX 75226 USA

## Abstract

Drugs that inhibit dipeptidyl peptidase-4 (DPP-4) are conventionally regarded as incretin-based agents that signal through the glucagon-like peptide-1 (GLP-1) receptor. However, inhibition of DPP-4 also potentiates the stem cell chemokine, stromal cell-derived factor-1 (SDF-1), which can promote inflammation, proliferative responses and neovascularization. In large-scale cardiovascular outcome trials, enhanced GLP-1 signaling has reduced the risk of atherosclerotic ischemic events, potentially because GLP-1 retards the growth and increases the stability of atherosclerotic plaques. However, DPP-4 inhibitors have not reduced the risk of major adverse cardiovascular events, possibly because potentiation of SDF-1 enhances plaque growth and instability, activates deleterious neurohormonal mechanisms, and promotes cardiac inflammation and fibrosis. Similarly, trials with GLP-1 agonists and sodium-glucose cotransporter 2 inhibitors have reported favorable effects on renal function, even after only 3–4 years of treatment. In contrast, no benefits on the rate of decline in glomerular filtration rate have been seen in trials of DPP-4 inhibitors, perhaps because the renal actions of DPP-4 inhibitors are primarily mediated by potentiation of SDF-1, not GLP-1. Experimentally, SDF-1 can promote podocyte injury and glomerulosclerosis. Furthermore, the natriuretic action of SDF-1 occurs primarily in the distal tubules, where it cannot utilize tubuloglomerular feedback to modulate the deleterious effects of glomerular hyperfiltration. Potentiation of SDF-1 in experimental models may also exacerbate both retinopathy and neuropathy. Therefore, although DPP-4 inhibitors have attractive clinical features, the benefits that might be expected from GLP-1 signaling may be undermined by their actions to enhance SDF-1.

## Background

Drugs that inhibit dipeptidyl peptidase-4 (DPP-4) are conventionally regarded as incretin-based agents that enhance the actions of endogenous gastrointestinal hormones (glucose-like peptide-1 [GLP-1] and glucose-dependent insulinotropic polypeptide) to promote the release of insulin from the pancreas [1, 2]. However, inhibition of DPP-4 also potentiates other substrates that are degraded by the enzyme, including several chemokines [3], particularly stromal cell-derived factor-1 (SDF-1) [4, 5]. This chemokine—also referred to as CXCL12 (C-X-C motif chemokine 12)—is responsible for the mobilization of hematopoietic stem and progenitor cells by signaling through its receptor CXCR4, and it contributes importantly to tissue inflammation, vascularity, repair and regeneration [6]. This function is defective in type 2 diabetes [7–9], presumably because DPP-4 activity is enhanced in patients with glucose intolerance [9–11].

## Potential role of stem-cell chemokines in type 2 diabetes

Experimentally, potentiation of SDF-1 can act to promote pancreatic β-cell genesis, differentiation and survival, and the chemokine may protect β cells from destruction as diabetes progresses [12, 13]. This chemokine may also play a protective role in the marshaling and recruitment of progenitor cells that could act to ameliorate ischemia, especially in peripheral limbs [14–17]. However, the ability of SDF-1 to promote repair involves both inflammation, angiogenesis and fibrosis, which could theoretically have adverse effects on the course of many of the macrovascular and microvascular complications of diabetes [18].

The gene for SDF-1 has been identified through genome-wide association studies as one of the key loci associated with increased susceptibility to coronary artery disease [19, 20]. Increased levels of SDF-1 are associated with increased severity of coronary artery obstructions [21], and high levels of the chemokine are seen in patients with an acute coronary syndrome and forebode a worse prognosis and an increased risk of heart failure [22–24]. SDF-1 may also play a critical role in the genesis of retinopathy, which starts with damage to small blood vessels in the eye but whose progression depends on a neovascular response that can be exacerbated by SDF-1 [18, 25]. Similarly, although SDF-1 may ameliorate kidney injury and promote repair after nondiabetic ischemia [26], potentiation of the chemokine can contribute to a proliferative response that leads to glomerulosclerosis, podocyte loss, and albuminuria [27, 28], thus implicating SDF-1 in the pathogenesis of diabetic nephropathy. Experimental studies have also identified SDF-1 as a mediator of pain and neovascularization in diabetic neuropathy [29, 30].

Despite their potential to potentiate SDF-1 and thereby exacerbate the vascular complications of type 2 diabetes, DPP-4 inhibitors have emerged as a popular choice for the treatment of the disease because of their ease of use, tolerability and ability to produce predictable and sustained lowering of blood glucose. Unlike older antidiabetic drugs [31–33], these drugs lower blood pressure and do not cause weight gain [34]; clinicians might expect such attributes to enhance the ability of these drugs to favorably modulate the risk of macrovascular and microvascular events [35, 36]. Furthermore, unlike long-acting GLP-1 analogs that also signal through the incretin pathway, DPP-4 inhibitors do not require parenteral administration, and their long-term use is associated with a low risk of gastrointestinal adverse effects and no meaningful increases in heart rate [37–40]. Use of these drugs is associated with a lower risk of hypoglycemia, when compared to insulin, sulfonylureas and thiazolidinediones [41, 42]. Additionally, unlike sodium-glucose transporter 2 (SGLT2) inhibitors, DPP-4 inhibitors do not adversely affect lipid metabolism or increase the risk of genitourinary infections [43]. The addition of DPP-4 inhibitors to patients already treated with metformin may seem particularly attractive, since both drugs may act to enhance circulating levels of GLP-1 and thus, may have synergistic effects on incretin receptor signaling [44–46].

However, the purpose of treating type 2 diabetes is not merely to lower levels of glycated hemoglobin, but to reduce the risk of the macrovascular and microvascular complications of the disease. Long-term outcomes trials with several different DPP-4 inhibitors have been performed, and their results are worth examining in order to understand both the mechanisms of action as well as the appropriate place of this class of drugs in diabetes care.

## Effect of DPP-4 inhibitors on macrovascular events in landmark trials

Four large-scale prospectively-designed cardiovascular outcomes trials have been carried out with DPP-4 inhibitors. A trial of alogliptin (EXAMINE) was performed in 5380 patients with an acute coronary syndrome; the median duration of follow-up was 1.5 years [47]. A trial of sitagliptin (TECOS) enrolled 14,735 patients who had clinically stable type 2 diabetes; the median duration of follow-up in the study was 3.0 years [48]. A trial of saxagliptin (SAVOR-TIMI53) studied 16,492 patients with diabetes who were followed for a median of 2.1 years [49]. A trial of omarigliptin evaluated 4202 diabetic patients without acute ischemic disease, who were followed for a median of 1.8 years, before early termination of the trial [50]. In all four trials, treatment with the DPP-4 inhibitor produced meaningful decreases in glycated hemoglobin.

Despite a sustained benefit on glycemic control, treatment with the four different DPP-4 inhibitors did not reduce the risk of major adverse cardiovascular events. In all four major trials, despite substantial statistical power to detect a clinically meaningful treatment effect, the administration of sitagliptin, saxagliptin, alogliptin and omarigliptin did not reduce the combined risk of cardiovascular death, non-fatal myocardial infarction and non-fatal stroke [47–50]. This lack of benefit stands in contrast with the significant or nearly-significant reduction in macrovascular risk reported with liraglutide, semaglutide and exenatide [51–53], which also enhance signaling through the GLP-1 receptor [54]. The findings with DPP-4 inhibitors also differ from the responses reported with SGLT2 inhibitors, which (in two trials) were reported to reduce major adverse cardiovascular events, especially the risk of new-onset heart failure [55, 56]. By comparison, the DPP-4 inhibitors, saxagliptin and alogliptin, carry a regulatory warning about the finding of an increased risk of hospitalization for heart failure in treated patients enrolled in large-scale trials carried out with these drugs [57].

The reasons for the lack of benefit of DPP-4 inhibitors on major cardiovascular outcomes could theoretically be related to the relatively short duration of follow-up in the landmark trials. However, this possibility seems unlikely, because over comparable periods of time, treatment with GLP-1 receptor agonists (which also enhance signaling through the GLP-1 pathway) reduced the risk of atherosclerotic ischemic events [51, 52]. Similarly, when given for treatment periods less than 4 years, SGLT2 inhibitors have had favorable effects on the risk of cardiovascular death, heart failure and nephropathy [55, 56, 58].

**Fig. 1:**
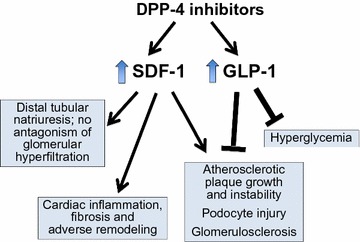
Effects of dipeptidyl peptidase-4 inhibition are mediated by potentiation of both stromal cell-derived factor-1 and glucagon-like peptide-1. Dipeptidyl peptidase-4 (DPP-4) inhibitors potentiate the actions of both stromal cell-derived factor-1 (SDF-1) and glucagon-like peptide-1 (GLP-1). These two peptides appear to exert opposing effects on atherosclerotic plaque growth and stability as well as on podocyte injury and glomerulosclerosis in the diabetic kidney. The effects of DPP-4 inhibitors on natriuresis as well as on cardiac inflammation, fibrosis and remodeling are primarily mediated by SDF-1

How then can these contrasting results be reconciled? In experimental studies, augmentation of the actions of GLP-1 retards the growth and increases the stability of atherosclerotic plaques, thus minimizing the likelihood of plaque rupture [59, 60]. Such an effect is mediated by an action of GLP-1 signaling to reduce the inflammatory response to vascular injury [59–62]. However, such a benefit might not be seen with DPP-4 inhibitors, since these drugs primarily potentiate levels of GLP-1 in the gastrointestinal tract and have modest effects on GLP-1 receptors in the systemic circulation [4]. Furthermore, DPP-4 inhibitors potentiate the actions of SDF-1 [5, 63], which acts as a proinflammatory chemokine to promote plaque growth and instability [2, 64–66]. The potentiation of endogenous SDF-1 by DPP-4 inhibitors could negate the benefits on atherosclerotic ischemic events that might be expected from enhanced GLP-1 signaling [59, 60] (Fig. 1).

Interestingly, SDF-1 can increase the number of circulating progenitor cells and direct the homing of stem cells to the heart in experimental myocardial injury [16, 17, 67–69]. However, in the absence of acute injury, SDF-1 signaling may impair cardiac contractility [70, 71]. Circulating levels of SDF-1 and the expression of receptors for SDF-1 are already increased in patients with heart failure, in the absence of DPP-4 inhibition [72, 73]. Further potentiation of SDF-1 could activate deleterious neurohormonal systems [74, 75], interact unfavorably with concurrently administered beta-adrenergic receptor blockers [71, 76], and promote progenitor cell infiltration, cardiac inflammation and adverse cardiac remodeling [77–79]. Is it possible that SDF-1 potentiation might explain the increased risk of heart failure reported in trials of DPP-4 inhibitors? [80, 81].

## Effect of DPP-4 inhibitors on microvascular events in landmark trials

An adequate assessment of the effect of antidiabetic drugs on the risk of microvascular events requires trials that evaluate durations of glucose-lowering treatments administered for a decade or longer. Interventions that lower blood glucose for 10 years have been shown to reduce the risk of retinopathy and nephropathy [82, 83]; changes in course of neuropathy may require more prolonged therapy. DPP-4 inhibitors have not been tested for such extended periods of time, and large-scale cardiovascular outcomes trials have not been designed to evaluate the effects of treatment with these drugs on microvascular risk.

Nevertheless, many of the large-scale cardiovascular trials have reported the effects of DPP-4 inhibitors on aspects of diabetic nephropathy, specifically changes in urinary protein excretion, in glomerular function over time, and in the risk of progression to end-stage renal disease [47, 48, 84]. In its large-scale trial, saxagliptin produced a sustained but modest effect on albuminuria [84]. Such an effect was not unexpected; hyperglycemia acts directly on glomerular podocytes to increase their permeability to albumin [85, 86], and its correction should reduce urinary protein excretion. However, a favorable effects of DPP-4 inhibitors on albuminuria has not been a consistent finding in clinical trials [87], possibly because potentiation of SDF-1 in podocytes may aggravate proteinuria, and thus, may oppose the benefits expected from glycemic control [28].

Despite an ability to reduce albuminuria, treatment with DPP-4 inhibitors has not been accompanied by beneficial changes in the clinical course of diabetic nephropathy. A meta-analysis of trials with linagliptin reported favorable effects on renal outcomes; however, the median duration of treatment was less than 6 months, and the benefit was driven primarily by a reduction in albuminuria [88]. In large-scale longer-term cardiovascular outcomes trials [47, 48, 84], long-term DPP-4 inhibition was associated with no change or a small decline in glomerular function that persisted during the entire duration of follow-up (up to 4 years). Furthermore, treatment with sitagliptin and saxagliptin did not diminish the risk of serious adverse renal events, as measured by a doubling of serum creatinine or the need for renal replacement therapy. In contrast, treatment with the GLP-1 receptor agonist liraglutide yielded a small improvement in kidney function at the end of the follow-up period [89], and in two large-scale trials, the use of SGLT2 inhibitors was accompanied by a meaningful and durable improvement in glomerular function and a reduction in the risk of serious adverse renal events [56, 58]. The advantages of SGLT2 inhibitors over DPP-4 inhibitors cannot be ascribed to differences in their antihyperglycemic effects, since in the large-scale trials, the two classes of drugs produced similar decreases in blood glucose during long-term treatment.

To the extent that hyperglycemia contributes to renal injury and nephropathy, the antihyperglycemic effects of long-term DPP-4 inhibition might be expected to slow the rate of decline of glomerular function, if this benefit can be sustained for prolonged periods of time [83, 84]. However, in the large-scale cardiovascular outcomes trials, the benefits of GLP-1 receptor agonists and SGLT2 inhibitors on renal function were seen relatively early in treatment, within only 3–4 years [56, 58]. Why did DPP-4 inhibitors not exert favorable renal effects when administered over these relatively short periods of time?

There are two possibilities. First, although the experimental data are conflicting [90], it is possible that potentiation of SDF-1 by DPP-4 inhibitors may enhance the inflammatory and proliferative responses to kidney injury and may thereby aggravate the course of diabetic nephropathy [27, 28]. This adverse effect may negate any benefit on renal function that might be achieved through GLP-1 receptor signaling [91, 92]. Second, kidney injury in diabetes appears to be related to glomerular hyperfiltration [93, 94], which is likely related to an excessive reabsorption of sodium in the proximal tubule, leading diminished delivery of sodium to the macula densa, and (via tubuloglomerular feedback) to afferent arteriolar dilatation [95, 96]. Both GLP-1 receptor agonists and SGLT2 inhibitors act directly on the proximal tubule to block sodium hyper-reabsorption [97, 98]; this effect, which is independent of their action on blood glucose, may underlie the early favorable actions on the kidney seen with these drugs in large-scale trials. In contrast, DPP-4 inhibitors exert a natriuretic effect by acting primarily on distal segments, apparently by enhancing the effects of SDF-1 [99] (Fig. 1). However, because this distal site of action that cannot utilize tubuloglomerular feedback to ameliorate glomerular hyperfiltration [95, 96], DPP-4 inhibition does not appear to exert early benefits on renal function in type 2 diabetes. If DPP-4 inhibitors exert any effect to inhibit sodium transport in the proximal tubule, this action does not appear to be mediated by potentiation of GLP-1 and or by pathways linked to the GLP-1 receptor [98–101]. The totality of evidence suggests that the effects of DPP-4 inhibitors on the kidney may be primarily mediated through potentiation of SDF-1 rather than of GLP-1.

## Summary and conclusions

DPP-4 inhibitors represent an attractive therapeutic option for the control of hyperglycemia in patients with type 2 diabetes due to their ease of use, tolerability and safety profile. However, unlike other newer antidiabetic drugs, DPP-4 inhibitors do not appear to exert meaningful effects on macrovascular or microvascular risk, at least when administered for periods of 3–4 years. This lack of early benefit may be related to the fact that the clinical profile of DPP-4 inhibitors may be dominated by the potentiation of endogenous peptides other than GLP-1 [1]. Many of the effects of DPP-4 inhibitors in large-scale clinical trials, including their effects on atherosclerotic ischemic events, heart failure, sodium excretion, albuminuria and glomerular function may be meaningfully influenced by their actions to enhance of the endogenous stem-cell chemokine, SDF-1. Only the antihyperglycemic effect of these drugs appears to be clearly related to the potentiation of incretins (i.e., GLP-1 and glucose-dependent insulinotropic polypeptide) [102, 103], possibly because such potentiation is confined to the gastrointestinal tract and may not be manifest systemically [63]. Given the complexity of the clinical effects exhibited by DPP-4 inhibitors in diabetes, it may not be entirely informative to refer to them solely as incretin-based drugs.

## References

[1] Andersen ES, Deacon CF, Holst JJ (2017). Do we know the true mechanism of action of the DPP-4 inhibitors?. Diabetes Obes Metab.

[2] Zhong J, Maiseyeu A, Davis SN, Rajagopalan S (2015). DPP4 in cardiometabolic disease: recent insights from the laboratory and clinical trials of DPP4 inhibition. Circ Res.

[3] Broxmeyer HE, Capitano M, Campbell TB, Hangoc G, Cooper S (2016). Modulation of hematopoietic chemokine effects in vitro and in vivo by DPP-4/CD26. Stem Cells Dev.

[4] Nauck M (2016). Incretin therapies: highlighting common features and differences in the modes of action of glucagon-like peptide-1 receptor agonists and dipeptidyl peptidase-4 inhibitors. Diabetes Obes Metab.

[5] Proost P, Struyf S, Schols D, Durinx C, Wuyts A, Lenaerts JP, De Clercq E, De Meester I, Van Damme J (1998). Processing by CD26/dipeptidyl-peptidase IV reduces the chemotactic and anti-HIV-1 activity of stromal-cell-derived factor-1alpha. FEBS Lett.

[6] Luo Q, Zhang B, Kuang D, Song G (2016). Role of stromal-derived factor-1 in mesenchymal stem cell paracrine-mediated tissue repair. Curr Stem Cell Res Ther.

[7] Fadini GP, Sartore S, Albiero M, Baesso I, Murphy E, Menegolo M, Grego F, de Kreutzenberg SV, Tiengo A, Agostini C, Avogaro A (2006). Number and function of endothelial progenitor cells as a marker of severity for diabetic vasculopathy. Arterioscler Thromb Vasc Biol.

[8] Ferraro F, Lymperi S, Méndez-Ferrer S, Saez B, Spencer JA, Yeap BY, Masselli E, Graiani G, Prezioso L, Rizzini EL, Mangoni M, Rizzoli V, Sykes SM, Lin CP, Frenette PS, Quaini F, Scadden DT (2011). Diabetes impairs hematopoietic stem cell mobilization by altering niche function. Sci Transl Med.

[9] Fadini GP, Albiero M, Seeger F, Poncina N, Menegazzo L, Angelini A, Castellani C, Thiene G, Agostini C, Cappellari R, Boscaro E, Zeiher A, Dimmeler S, Avogaro A (2013). Stem cell compartmentalization in diabetes and high cardiovascular risk reveals the role of DPP-4 in diabetic stem cell mobilopathy. Basic Res Cardiol.

[10] Mannucci E, Pala L, Ciani S, Bardini G, Pezzatini A, Sposato I, Cremasco F, Ognibene A, Rotella CM (2005). Hyperglycaemia increases dipeptidyl peptidase IV activity in diabetes mellitus. Diabetologia.

[11] Ryskjaer J, Deacon CF, Carr RD, Krarup T, Madsbad S, Holst J, Vilsbøll T (2006). Plasma dipeptidyl peptidase-IV activity in patients with type-2 diabetes mellitus correlates positively with HbAlc levels, but is not acutely affected by food intake. Eur J Endocrinol.

[12] Kayali AG, Lopez AD, Hao E, Hinton A, Hayek A, King CC (2012). The SDF-1α/CXCR4 axis is required for proliferation and maturation of human fetal pancreatic endocrine progenitor cells. PLoS ONE.

[13] Liu Z, Stanojevic V, Avadhani S, Yano T, Habener JF (2011). Stromal cell-derived factor-1 (SDF-1)/chemokine (C-X-C motif) receptor 4 (CXCR4) axis activation induces intra-islet glucagon-like peptide-1 (GLP-1) production and enhances beta cell survival. Diabetologia.

[14] Dai X, Yan X, Zeng J, Chen J, Wang Y, Chen J, Li Y, Barati MT, Wintergerst KA, Pan K, Nystoriak MA, Conklin DJ, Rokosh G, Epstein PN, Li X, Tan Y (2017). Elevating CXCR7 improves angiogenic function of EPCs via Akt/GSK-3β/Fyn-mediated Nrf2 activation in diabetic limb ischemia. Circ Res.

[15] Fiordaliso F, Maggioni S, Balconi G, Schiarea S, Corbelli A, De Luigi A, Figliuzzi M, Antoniou X, Chiabrando C, Masson S, Cervo L, Latini R (2016). Effects of dipeptidyl peptidase-4 (DPP-4) inhibition on angiogenesis and hypoxic injury in type 2 diabetes. Life Sci.

[16] Shih CM, Chen YH, Lin YW, Tsao NW, Wu SC, Kao YT, Chiang KH, Li CY, Chang NC, Lin CY, Huang CY, Lin FY (2014). MK-0626, a dipeptidyl peptidase-4 inhibitor, improves neovascularization by increasing both the number of circulating endothelial progenitor cells and endothelial nitric oxide synthetase expression. Curr Med Chem.

[17] Dei Cas A, Spigoni V, Cito M, Aldigeri R, Ridolfi V, Marchesi E, Marina M, Derlindati E, Aloe R, Bonadonna RC, Zavaroni I (2017). Vildagliptin, but not glibenclamide, increases circulating endothelial progenitor cell number: a 12-month randomized controlled trial in patients with type 2 diabetes. Cardiovasc Diabetol.

[18] Vidaković M, Grdović N, Dinić S, Mihailović M, Uskoković A, Jovanović JA (2015). The importance of the CXCL12/CXCR4 axis in therapeutic approaches to diabetes mellitus attenuation. Front Immunol.

[19] Samani NJ, Erdmann J, Hall AS, Hengstenberg C, Mangino M, Mayer B, Dixon RJ, Meitinger T, Braund P, Wichmann HE, Barrett JH, König IR, Stevens SE, Szymczak S, Tregouet DA, Iles MM, Pahlke F, Pollard H, Lieb W, Cambien F, Fischer M, Ouwehand W, Blankenberg S, Balmforth AJ, Baessler A, Ball SG, Strom TM, Braenne I, Gieger C, Deloukas P, Tobin MD, Ziegler A, Thompson JR, Schunkert H, WTCCC and the Cardiogenics Consortium (2007). Genomewide association analysis of coronary artery disease. N Engl J Med.

[20] Farouk SS, Rader DJ, Reilly MP, Mehta NN (2010). CXCL12: a new player in coronary disease identified through human genetics. Trends Cardiovasc Med.

[21] Ferdousie VT, Mohammadi MM, Hassanshahi G, Khorramdelazad H, Falahati-Pour SK, Mirzaei M, Tavakoli MA, Kamiab Z, Ahmadi Z, Vazirinejad R, Shahrabadi E, Koniari I, Kounis NG, Esmaeili Nadimi A (2017). Serum CXCL10 and CXCL12 chemokine levels are associated with the severity of coronary artery disease and coronary artery occlusion. Int J Cardiol.

[22] Rath D, Chatterjee M, Borst O, Müller K, Stellos K, Mack AF, Bongartz A, Bigalke B, Langer H, Schwab M, Gawaz M, Geisler T (2014). Expression of stromal cell-derived factor-1 receptors CXCR4 and CXCR7 on circulating platelets of patients with acute coronary syndrome and association with left ventricular functional recovery. Eur Heart J.

[23] Ghasemzadeh N, Hritani AW, De Staercke C, Eapen DJ, Veledar E, Al Kassem H, Khayata M, Zafari AM, Sperling L, Hooper C, Vaccarino V, Mavromatis K, Quyyumi AA (2015). Plasma stromal cell-derived factor 1α/CXCL12 level predicts long-term adverse cardiovascular outcomes in patients with coronary artery disease. Atherosclerosis.

[24] Subramanian S, Liu C, Aviv A, Ho JE, Courchesne P, Muntendam P, Larson MG, Cheng S, Wang TJ, Mehta NN, Levy D (2014). Stromal cell-derived factor 1 as a biomarker of heart failure and mortality risk. Arterioscler Thromb Vasc Biol.

[25] Butler JM, Guthrie SM, Koc M, Afzal A, Caballero S, Brooks HL, Mames RN, Segal MS, Grant MB, Scott EW (2005). SDF-1 is both necessary and sufficient to promote proliferative retinopathy. J Clin Invest.

[26] Liu H, Liu S, Li Y, Wang X, Xue W, Ge G, Luo X (2012). The role of SDF-1-CXCR4/CXCR7 axis in the therapeutic effects of hypoxia-preconditioned mesenchymal stem cells for renal ischemia/reperfusion injury. PLoS ONE.

[27] Darisipudi MN, Kulkarni OP, Sayyed SG, Ryu M, Migliorini A, Sagrinati C, Parente E, Vater A, Eulberg D, Klussmann S, Romagnani P, Anders HJ (2011). Dual blockade of the homeostatic chemokine CXCL12 and the proinflammatory chemokine CCL2 has additive protective effects on diabetic kidney disease. Am J Pathol.

[28] Sayyed SG, Hägele H, Kulkarni OP, Endlich K, Segerer S, Eulberg D, Klussmann S, Anders HJ (2009). Podocytes produce homeostatic chemokine stromal cell-derived factor-1/CXCL12, which contributes to glomerulo-sclerosis, podocyte loss and albuminuria in a mouse model of type 2 diabetes. Diabetologia.

[29] Menichella DM, Abdelhak B, Ren D, Shum A, Frietag C, Miller RJ (2014). CXCR4 chemokine receptor signaling mediates pain in diabetic neuropathy. Mol Pain..

[30] Kim BJ, Lee JK, Schuchman EH, Jin HK, Bae JS (2013). Synergistic vasculogenic effects of AMD3100 and stromal-cell-derived factor-1α in vasa nervorum of the sciatic nerve of mice with diabetic peripheral neuropathy. Cell Tissue Res.

[31] Manhiani MM, Cormican MT, Brands MW (2011). Chronic sodium-retaining action of insulin in diabetic dogs. Am J Physiol Renal Physiol.

[32] Bełtowski J, Rachańczyk J, Włodarczyk M (2013). Thiazolidinedione-induced fluid retention: recent insights into the molecular mechanisms. PPAR Res.

[33] Gerstein HC, Bosch J, Dagenais GR, Díaz R, Jung H, Maggioni AP, Pogue J, Probstfield J, Ramachandran A, Riddle MC, Rydén LE, Yusuf S, ORIGIN Trial Investigators (2012). Basal insulin and cardiovascular and other outcomes in dysglycemia. N Engl J Med.

[34] Nauck MA, Meier JJ, Cavender MA, El Aziz MA, Drucker DJ (2017). Cardiovascular actions and clinical outcomes with glucagon-like peptide-1 receptor agonists and dipeptidyl peptidase-4 inhibitors. Circulation.

[35] UK Prospective Diabetes Study Group (1998). Tight blood pressure control and risk of macrovascular and microvascular complications in type 2 diabetes: UKPDS 38. BMJ.

[36] Xie X, Atkins E, Lv J, Bennett A, Neal B, Ninomiya T, Woodward M, MacMahon S, Turnbull F, Hillis GS, Chalmers J, Mant J, Salam A, Rahimi K, Perkovic V, Rodgers A (2016). Effects of intensive blood pressure lowering on cardiovascular and renal outcomes: updated systematic review and meta-analysis. Lancet.

[37] Deacon CF, Mannucci E, Ahrén B (2012). Glycaemic efficacy of glucagon-like peptide-1 receptor agonists and dipeptidyl peptidase-4 inhibitors as add-on therapy to metformin in subjects with type 2 diabetes-a review and meta analysis. Diabetes Obes Metab.

[38] Maruthur NM, Tseng E, Hutfless S, Wilson LM, Suarez-Cuervo C, Berger Z, Chu Y, Iyoha E, Segal JB, Bolen S (2016). Diabetes medications as monotherapy or metformin-based combination therapy for type 2 diabetes: a systematic review and meta-analysis. Ann Intern Med.

[39] Wang T, Gou Z, Wang F, Ma M, Zhai SD (2014). Comparison of GLP-1 analogues versus sitagliptin in the management of type 2 diabetes: systematic review and meta-analysis of head-to-head studies. PLoS ONE.

[40] Lorenz M, Lawson F, Owens D, Raccah D, Roy-Duval C, Lehmann A, Perfetti R, Blonde L (2017). Differential effects of glucagon-like peptide-1 receptor agonists on heart rate. Cardiovasc Diabetol.

[41] Nyström T, Bodegard J, Nathanson D, Thuresson M, Norhammar A, Eriksson JW (2017). Second line initiation of insulin compared with DPP-4 inhibitors after metformin monotherapy is associated with increased risk of all-cause mortality, cardiovascular events, and severe hypoglycemia. Diabetes Res Clin Pract.

[42] Palmer SC, Mavridis D, Nicolucci A, Johnson DW, Tonelli M, Craig JC, Maggo J, Gray V, De Berardis G, Ruospo M, Natale P, Saglimbene V, Badve SV, Cho Y, Nadeau-Fredette AC, Burke M, Faruque L, Lloyd A, Ahmad N, Liu Y, Tiv S, Wiebe N, Strippoli GF (2016). Comparison of clinical outcomes and adverse events associated with glucose-lowering drugs in patients with type 2 diabetes: a meta-analysis. JAMA.

[43] Cha SA, Park YM, Yun JS, Lim TS, Song KH, Yoo KD, Ahn YB, Ko SH (2017). A comparison of effects of DPP-4 inhibitor and SGLT2 inhibitor on lipid profile in patients with type 2 diabetes. Lipids Health Dis.

[44] Yasuda N, Inoue T, Nagakura T, Yamazaki K, Kira K, Saeki T, Tanaka I (2002). Enhanced secretion of glucagon-like peptide 1 by biguanide compounds. Biochem Biophys Res Commun.

[45] Wu T, Thazhath SS, Bound MJ, Jones KL, Horowitz M, Rayner CK (2014). Mechanism of increase in plasma intact GLP-1 by metformin in type 2 diabetes: stimulation of GLP-1 secretion or reduction in plasma DPP-4 activity?. Diabetes Res Clin Pract.

[46] Preiss D, Dawed A, Welsh P, Heggie A, Jones AG, Dekker J, Koivula R, Hansen TH, Stewart C, Holman RR, Franks PW, Walker M, Pearson ER, Sattar N, DIRECT consortium group (2017). Sustained influence of metformin therapy on circulating glucagon-like peptide-1 levels in individuals with and without type 2 diabetes. Diabetes Obes Metab.

[47] White WB, Cannon CP, Heller SR, Nissen SE, Bergenstal RM, Bakris GL, Perez AT, Fleck PR, Mehta CR, Kupfer S, Wilson C, Cushman WC, Zannad F, EXAMINE Investigators (2013). Alogliptin after acute coronary syn-drome in patients with type 2 diabetes. N Engl J Med.

[48] Green JB, Bethel MA, Armstrong PW, Buse JB, Engel SS, Garg J, Josse R, Kaufman KD, Koglin J, Korn S, Lachin JM, McGuire DK, Pencina MJ, Standl E, Stein PP, Suryawanshi S, Van de Werf F, Peterson ED, Holman RR, TECOS Study Group (2015). Effect of sitagliptin on cardiovascular outcomes in type 2 diabetes. N Engl J Med.

[49] Scirica BM, Bhatt DL, Braunwald E, Steg PG, Davidson J, Hirshberg B, Ohman P, Frederich R, Wiviott SD, Hoffman EB, Cavender MA, Udell JA, Desai NR, Mosenzon O, McGuire DK, Ray KK, Leiter LA, Raz I, SAVOR-TIMI 53 Steering Committee and Investigators (2013). Saxagliptin and cardiovascular outcomes in patients with type 2 diabetes mellitus. N Engl J Med.

[50] Gantz I, Chen M, Suryawanshi S, Ntabadde C, Shah S, O’Neill EA, Engel SS, Kaufman KD, Lai E (2017). A randomized, placebo-controlled study of the cardiovascular safety of the once-weekly DPP-4 inhibitor omarigliptin in patients with type 2 diabetes mellitus. Cardiovasc Diabetol.

[51] Marso SP, Daniels GH, Brown-Frandsen K, Kristensen P, Mann JF, Nauck MA, Nissen SE, Pocock S, Poulter NR, Ravn LS, Steinberg WM, Stockner M, Zinman B, Bergenstal RM, Buse JB, LEADER Steering Committee; LEADER Trial Investigators (2016). Liraglutide and cardiovascular outcomes in type 2 diabetes. N Engl J Med.

[52] Marso SP, Bain SC, Consoli A, Eliaschewitz FG, Jódar E, Leiter LA, Lingvay I, Rosenstock J, Seufert J, Warren ML, Woo V, Hansen O, Holst AG, Pettersson J, Vilsbøll T, SUSTAIN-6 Investigators (2016). Semaglutide and cardiovascular outcomes in patients with type 2 diabetes. N Engl J Med.

[53] Holman RR, Bethel MA, Mentz RJ, Thompson VP, Lokhnygina Y, Buse JB, Chan JC, Choi J, Gustavson SM, Iqbal N, Maggioni AP, Marso SP, Öhman P, Pagidipati NJ, Poulter N, Ramachandran A, Zinman B, Hernandez AF, EXSCEL Study Group (2017). Effects of once-weekly exenatide on cardiovascular outcomes in type 2 diabetes. N Engl J Med.

[54] Zhang Z, Chen X, Lu P, Zhang J, Xu Y, He W, Li M, Zhang S, Jia J, Shao S, Xie J, Yang Y, Yu X (2017). Incretin-based agents in type 2 diabetic patients at cardiovascular risk: compare the effect of GLP-1 agonists and DPP-4 inhibitors on cardiovascular and pancreatic outcomes. Cardiovasc Diabetol.

[55] Zinman B, Wanner C, Lachin JM, Fitchett D, Bluhmki E, Hantel S, Mattheus M, Devins T, Johansen OE, Woerle HJ, Broedl UC, Inzucchi SE, EMPA-REG OUTCOME Investigators (2015). Empagliflozin, cardiovascular out-comes, and mortality in type 2 diabetes. N Engl J Med.

[56] Neal B, Perkovic V, Mahaffey KW, de Zeeuw D, Fulcher G, Erondu N, Shaw W, Law G, Desai M, Matthews DR, CANVAS Program Collaborative Group (2017). Canagliflozin and cardiovascular and renal events in type 2 diabetes. N Engl J Med.

[57] US Food and Drug Administration. Diabetes medications containing saxagliptin and alogliptin: drug safety communication—risk of heart failure. https://www.fda.gov/safety/medwatch/safetyinformation/safetyalertsforhumanmedicalproducts/ucm494252.htm. Accessed 5 Apr 2016.

[58] Wanner C, Inzucchi SE, Lachin JM, Fitchett D, von Eynatten M, Mattheus M, Johansen OE, Woerle HJ, Broedl UC, Zinman B, EMPA-REG OUTCOME Investigators (2016). Empagliflozin and progression of kidney disease in type 2 diabetes. N Engl J Med.

[59] Sudo M, Li Y, Hiro T, Takayama T, Mitsumata M, Shiomi M, Sugitani M, Matsumoto T, Hao H, Hirayama A (2017). Inhibition of plaque progression and promotion of plaque stability by glucagon-like peptide-1 receptor agonist: serial in vivo findings from iMap-IVUS in Watanabe heritable hyperlipidemic rabbits. Atherosclerosis.

[60] Vinué Á, Navarro J, Herrero-Cervera A, García-Cubas M, Andrés-Blasco I, Martínez-Hervás S, Real JT, Ascaso JF, González-Navarro H (2017). The GLP-1 analogue lixisenatide decreases atherosclerosis in insulin-resistant mice by modulating macrophage phenotype. Diabetologia.

[61] Burgmaier M, Liberman A, Möllmann J, Kahles F, Reith S, Lebherz C, Marx N, Lehrke M (2013). Glucagon-like peptide-1 (GLP-1) and its split products GLP-1(9-37) and GLP-1(28-37) stabilize atherosclerotic lesions in apoe^−/−^ mice. Atherosclerosis.

[62] Gaspari T, Welungoda I, Widdop RE, Simpson RW, Dear AE (2013). The GLP-1 receptor agonist liraglutide inhibits progression of vascular disease via effects on atherogenesis, plaque stability and endothelial function in an ApoE(−/−) mouse model. Diab Vasc Dis Res.

[63] Broxmeyer HE, Hoggatt J, O’Leary HA, Mantel C, Chitteti BR, Cooper S, Messina-Graham S, Hangoc G, Farag S, Rohrabaugh SL, Ou X, Speth J, Pelus LM, Srour EF, Campbell TB (2012). Dipeptidylpeptidase 4 negatively regulates colony-stimulating factor activity and stress hematopoiesis. Nat Med.

[64] Kodali R, Hajjou M, Berman AB, Bansal MB, Zhang S, Pan JJ, Schecter AD (2006). Chemokines induce matrix metalloproteinase-2 through activation of epidermal growth factor receptor in arterial smooth muscle cells. Cardiovasc Res.

[65] Ma W, Liu Y, Ellison N, Shen J (2013). Induction of C-X-C chemokine receptor type 7 (CXCR7) switches stromal cell-derived factor-1 (SDF-1) signaling and phagocytic activity in macrophages linked to atherosclerosis. J Biol Chem.

[66] Zernecke A, Schober A, Bot I, von Hundelshausen P, Liehn EA, Möpps B, Mericskay M, Gierschik P, Biessen EA, Weber C (2005). SDF-1alpha/CXCR4 axis is instrumental in neointimal hyperplasia and recruitment of smooth muscle progenitor cells. Circ Res.

[67] Anderluh M, Kocic G, Tomovic K, Kocic R, Deljanin-Ilic M, Smelcerovic A (2016). Cross-talk between the dipeptidyl peptidase-4 and stromal cell-derived factor-1 in stem cell homing and myocardial repair: potential impact of dipeptidyl peptidase-4 inhibitors. Pharmacol Ther.

[68] Kubota A, Takano H, Wang H, Hasegawa H, Tadokoro H, Hirose M, Kobara Y, Yamada-Inagawa T, Komuro I, Kobayashi Y (2016). DPP-4 inhibition has beneficial effects on the heart after myocardial infarction. J Mol Cell Cardiol.

[69] Connelly KA, Advani A, Zhang Y, Advani SL, Kabir G, Abadeh A, Desjardins JF, Mitchell M, Thai K, Gilbert RE (2016). Dipeptidyl peptidase-4 inhibition improves cardiac function in experimental myocardial infarction: role of stromal cell-derived factor-1α. J Diabetes.

[70] Pyo RT, Sui J, Dhume A, Palomeque J, Blaxall BC, Diaz G, Tunstead J, Logothetis DE, Hajjar RJ, Schecter AD (2006). CXCR4 modulates contractility in adult cardiac myocytes. J Mol Cell Cardiol.

[71] LaRocca TJ, Schwarzkopf M, Altman P, Zhang S, Gupta A, Gomes I, Alvin Z, Champion HC, Haddad G, Hajjar RJ, Devi LA, Schecter AD, Tarzami ST (2010). β2-Adrenergic receptor signaling in the cardiac myocyte is modulated by interactions with CXCR4. J Cardiovasc Pharmacol.

[72] Damås JK, Eiken HG, Oie E, Bjerkeli V, Yndestad A, Ueland T, Tonnessen T, Geiran OR, Aass H, Simonsen S, Christensen G, Froland SS, Attramadal H, Gullestad L, Aukrust P (2000). Myocardial expression of CC- and CXC-chemokines and their receptors in human end-stage heart failure. Cardiovasc Res.

[73] Aukrust P, Ueland T, Müller F, Andreassen AK, Nordøy I, Aas H, Kjekshus J, Simonsen S, Frøland SS, Gullestad L (1998). Elevated circulating levels of C–C chemokines in patients with congestive heart failure. Circulation.

[74] Wei SG, Zhang ZH, Yu Y, Felder RB (2014). Central SDF-1/CXCL12 expression and its cardiovascular and sympathetic effects: the role of angiotensin II, TNF-α, and MAP kinase signaling. Am J Physiol Heart Circ Physiol.

[75] Wei SG, Zhang ZH, Yu Y, Weiss RM, Felder RB (2012). Central actions of the chemokine stromal cell-derived factor 1 contribute to neurohumoral excitation in heart failure rats. Hypertension.

[76] Miyoshi T, Nakamura K, Yoshida M, Miura D, Oe H, Akagi S, Sugiyama H, Akazawa K, Yonezawa T, Wada J, Ito H (2014). Effect of vildagliptin, a dipeptidyl peptidase 4 inhibitor, on cardiac hypertrophy induced by chronic beta-adrenergic stimulation in rats. Cardiovasc Diabetol.

[77] Mühlstedt S, Ghadge SK, Duchene J, Qadri F, Järve A, Vilianovich L, Popova E, Pohlmann A, Niendorf T, Boyé P, Özcelik C, Bader M (2016). Cardiomyocyte-derived CXCL12 is not involved in cardiogenesis but plays a crucial role in myocardial infarction. J Mol Med (Berl).

[78] Uematsu M, Yoshizaki T, Shimizu T, Obata JE, Nakamura T, Fujioka D, Watanabe K, Watanabe Y, Kugiyama K (2015). Sustained myocardial production of stromal cell-derived factor-1α was associated with left ventricular adverse remodeling in patients with myocardial infarction. Am J Physiol Heart Circ Physiol.

[79] Chu PY, Walder K, Horlock D, Williams D, Nelson E, Byrne M, Jandeleit-Dahm K, Zimmet P, Kaye DM (2015). CXCR4 antagonism attenuates the development of diabetic cardiac fibrosis. PLoS ONE.

[80] Verma S, Goldenberg RM, Bhatt DL, Farkouh ME, Quan A, Teoh H, Connelly KA, Leiter LA, Friedrich JO (2017). Dipeptidyl peptidase-4 inhibitors and the risk of heart failure: a systematic review and meta-analysis. CMAJ Open.

[81] Li L, Li S, Deng K, Liu J, Vandvik PO, Zhao P, Zhang L, Shen J, Bala MM, Sohani ZN, Wong E, Busse JW, Ebrahim S, Malaga G, Rios LP, Wang Y, Chen Q, Guyatt GH, Sun X (2016). Dipeptidyl peptidase-4 inhibitors and risk of heart failure in type 2 diabetes: systematic review and meta-analysis of randomised and observational studies. BMJ.

[82] UK Prospective Diabetes Study (UKPDS) Group (1998). Intensive blood-glucose control with sulphonylureas or insulin compared with conventional treatment and risk of complications in patients with type 2 diabetes (UKPDS 33). Lancet.

[83] DCCT/EDIC research group (2014). Effect of intensive diabetes treatment on albuminuria in type 1 diabetes: long-term follow-up of the diabetes control and complications trial and epidemiology of diabetes interventions and complications study. Lancet Diabetes Endocrinol.

[84] Mosenzon O, Leibowitz G, Bhatt DL, Cahn A, Hirshberg B, Wei C, Im K, Rozenberg A, Yanuv I, Stahre C, Ray KK, Iqbal N, Braunwald E, Scirica BM, Raz I (2017). Effect of saxagliptin on renal outcomes in the SAVOR-TIMI 53 trial. Diabetes Care.

[85] Swärd P, Rippe B (2012). Acute and sustained actions of hyperglycaemia on endothelial and glomerular barrier permeability. Acta Physiol (Oxf).

[86] Piwkowska A, Rogacka D, Audzeyenka I, Angielski S, Jankowski M (2014). High glucose increases glomerular filtration barrier permeability by activating protein kinase G type Iα subunits in a Nox4-dependent manner. Exp Cell Res.

[87] Groop PH, Cooper ME, Perkovic V, Hocher B, Kanasaki K, Haneda M, Schernthaner G, Sharma K, Stanton RC, Toto R, Cescutti J, Gordat M, Meinicke T, Koitka-Weber A, Thiemann S, von Eynatten M (2017). Linagliptin and its effects on hyperglycaemia and albuminuria in patients with type 2 diabetes and renal dysfunction: the randomized MARLINA-T2D trial. Diabetes Obes Metab.

[88] Cooper ME, Perkovic V, McGill JB, Groop PH, Wanner C, Rosenstock J, Hehnke U, Woerle HJ, von Eynatten M (2015). Kidney disease end points in a pooled analysis of individual patient-level data from a large clinical trials program of the dipeptidyl peptidase 4 inhibitor linagliptin in type 2 diabetes. Am J Kidney Dis.

[89] Mann JFE, Ørsted DD, Brown-Frandsen K, Marso SP, Poulter NR, Rasmussen S, Tornøe K, Zinman B, Buse JB, LEADER Steering Committee and Investigators (2017). Liraglutide and renal outcomes in type 2 diabetes. N Engl J Med.

[90] Takashima S, Fujita H, Fujishima H, Shimizu T, Sato T, Morii T, Tsukiyama K, Narita T, Takahashi T, Drucker DJ, Seino Y, Yamada Y (2016). Stromal cell-derived factor-1 is upregulated by dipeptidyl peptidase-4 inhibition and has protective roles in progressive diabetic nephropathy. Kidney Int.

[91] Muskiet MHA, Tonneijck L, Smits MM, van Baar MJB, Kramer MHH, Hoorn EJ, Joles JA, van Raalte DH (2017). GLP-1 and the kidney: from physiology to pharmacology and outcomes in diabetes. Nat Rev Nephrol.

[92] Dicembrini I, Nreu B, Scatena A, Andreozzi F, Sesti G, Mannucci E, Monami M (2017). Microvascular effects of glucagon-like peptide-1 receptor agonists in type 2 diabetes: a meta-analysis of randomized controlled trials. Acta Diabetol.

[93] Stackhouse S, Miller PL, Park SK, Meyer TW (1990). Reversal of glomerular hyperfiltration and renal hypertrophy by blood glucose normalization in diabetic rats. Diabetes.

[94] Tonneijck L, Muskiet MH, Smits MM, van Bommel EJ, Heerspink HJ, van Raalte DH, Joles JA (2017). Glomerular hyperfiltration in diabetes: mechanisms, clinical significance, and treatment. J Am Soc Nephrol.

[95] Hallow KM, Gebremichael Y, Helmlinger G, Vallon V (2017). Primary proximal tubule hyperreabsorption and impaired tubular transport counterregulation determine glomerular hyperfiltration in diabetes: a modeling analysis. Am J Physiol Renal Physiol.

[96] Layton AT, Vallon V, Edwards A (2015). Modeling oxygen consumption in the proximal tubule: effects of NHE and SGLT2 inhibition. Am J Physiol Renal Physiol.

[97] Packer M, Anker SD, Butler J, Filippatos G, Zannad F (2017). Effects of sodium-glucose cotransporter 2 inhibitors for the treatment of patients with heart failure: proposal of a novel mechanism of action. JAMA Cardiol.

[98] Skov J, Pedersen M, Holst JJ, Madsen B, Goetze JP, Rittig S, Jonassen T, Frøkiaer J, Dejgaard A, Christiansen JS (2016). Short-term effects of liraglutide on kidney function and vasoactive hormones in type 2 diabetes: a randomized clinical trial. Diabetes Obes Metab.

[99] Lovshin JA, Rajasekeran H, Lytvyn Y, Lovblom LE, Khan S, Alemu R, Locke A, Lai V, He H, Hittle L, Wang W, Drucker DJ, Cherney DZI (2017). Dipeptidyl peptidase 4 inhibition stimulates distal tubular natriuresis and increases in circulating SDF-1α1-67 in patients with type 2 diabetes. Diabetes Care.

[100] Rieg T, Gerasimova M, Murray F, Masuda T, Tang T, Rose M, Drucker DJ, Vallon V (2012). Natriuretic effect by exendin-4, but not the DPP-4 inhibitor alogliptin, is mediated via the GLP-1 receptor and preserved in obese type 2 diabetic mice. Am J Physiol Renal Physiol.

[101] Girardi AC, Knauf F, Demuth HU, Aronson PS (2004). Role of dipeptidyl peptidase IV in regulating activity of Na+/H+ exchanger isoform NHE3 in proximal tubule cells. Am J Physiol Cell Physiol.

[102] Nauck MA, Kahle M, Baranov O, Deacon CF, Holst JJ (2017). Addition of a dipeptidyl peptidase-4 inhibitor, sitagliptin, to ongoing therapy with the glucagon-like peptide-1 receptor agonist liraglutide: a randomized controlled trial in patients with type 2 diabetes. Diabetes Obes Metab.

[103] Hansotia T, Baggio LL, Delmeire D, Hinke SA, Yamada Y, Tsukiyama K, Seino Y, Holst JJ, Schuit F, Drucker DJ (2004). Double incretin receptor knockout (DIRKO) mice reveal an essential role for the enteroinsular axis in transducing the glucoregulatory actions of DPP-IV inhibitors. Diabetes.

